# 3D hanging spheroid plate for high-throughput CAR T cell cytotoxicity assay

**DOI:** 10.1186/s12951-021-01213-8

**Published:** 2022-01-10

**Authors:** Zhenzhong Chen, Seokgyu Han, Arleen Sanny, Dorothy Leung-Kwan Chan, Danny van Noort, Wanyoung Lim, Andy Hee-Meng Tan, Sungsu Park

**Affiliations:** 1 School of Mechanical Engineering, Sungkyunkwan University (SKKU), Suwon, 16419 South Korea; 2 Bioprocessing Technology Institute (BTI), Agency for Science, Technology and Research (A*STAR), Singapore, 138668 Singapore; 3 Centro de Investigación en Bioingeniería, Universidad de Ingenieria y Tecnologia - UTEC, Lima 04, Peru; 4 Biotechnology, Linköping University, SE-581 83 Linköping, Sweden; 5 Department of Biomedical Engineering, Sungkyunkwan University (SKKU), Suwon, 16419 South Korea; 6 Institute of Quantum Biophysics (IQB), Sungkyunkwan University (SKKU), Suwon, 16419 South Korea

**Keywords:** HER2-CAR T cell, 3D hanging spheroid plate, Tumor spheroid, Cytotoxicity assay

## Abstract

**Background:**

Most high-throughput screening (HTS) systems studying the cytotoxic effect of chimeric antigen receptor (CAR) T cells on tumor cells rely on two-dimensional cell culture that does not recapitulate the tumor microenvironment (TME). Tumor spheroids, however, can recapitulate the TME and have been used for cytotoxicity assays of CAR T cells. But a major obstacle to the use of tumor spheroids for cytotoxicity assays is the difficulty in separating unbound CAR T and dead tumor cells from spheroids. Here, we present a three-dimensional hanging spheroid plate (3DHSP), which facilitates the formation of spheroids and the separation of unbound and dead cells from spheroids during cytotoxicity assays.

**Results:**

The 3DHSP is a 24-well plate, with each well composed of a hanging dripper, spheroid wells, and waste wells. In the dripper, a tumor spheroid was formed and mixed with CAR T cells. In the 3DHSP, droplets containing the spheroids were deposited into the spheroid separation well, where unbound and dead T and tumor cells were separated from the spheroid through a gap into the waste well by tilting the 3DHSP by more than 20°. Human epidermal growth factor receptor 2 (HER2)-positive tumor cells (BT474 and SKOV3) formed spheroids of approximately 300–350 μm in diameter after 2 days in the 3DHSP. The cytotoxic effects of T cells engineered to express CAR recognizing HER2 (HER2-CAR T cells) on these spheroids were directly measured by optical imaging, without the use of live/dead fluorescent staining of the cells. Our results suggest that the 3DHSP could be incorporated into a HTS system to screen for CARs that enable T cells to kill spheroids formed from a specific tumor type with high efficacy or for spheroids consisting of tumor types that can be killed efficiently by T cells bearing a specific CAR.

**Conclusions:**

The results suggest that the 3DHSP could be incorporated into a HTS system for the cytotoxic effects of CAR T cells on tumor spheroids.

**Graphical Abstract:**

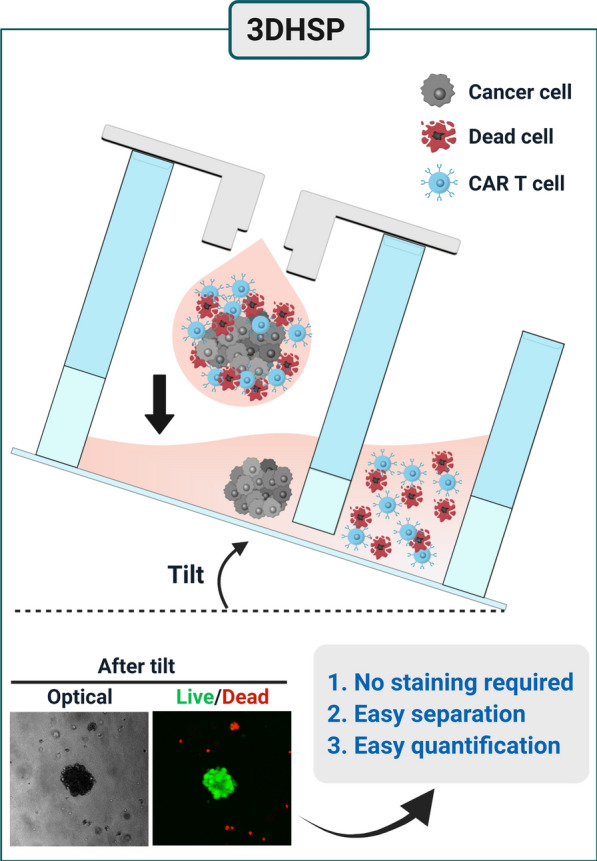

**Supplementary Information:**

The online version contains supplementary material available at 10.1186/s12951-021-01213-8.

## Background

Chimeric antigen receptor (CAR) T cells are T cells genetically modified to recognize specific surface antigens on tumor cells [1, 2]. CAR T cell therapies, notably therapies targeting cluster of differentiation (CD)-19, have been approved by the U.S. Food and Drug Administration (FDA) for the treatment of B-cell malignancies, against which they have been proven to exert highly potent therapeutic effects [3, 4]. Much effort has been focused on the development of high-throughput screening (HTS) systems to select CAR T cells that can bind to tumor cells [5]. However, most of the monolayer cell culture of these HTS systems [6] does not recapitulate three-dimensional (3D) tumor microenvironment (TME) in vivo [7, 8], and therefore, have limitations in accurately interrogating the CAR T cytotoxicity in vivo.

TME is extremely complex and composed of various cell types, including tumor cells and non-cellular components, such as extracellular matrix [9, 10]. Some physiological factors in TME can be recapitulated by spherical aggregates of tumor cells, i.e., tumor spheroids [11, 12]. Tumor spheroids can have a hypoxic core and generate a gradient of immunosuppressive cytokines, such as interleukin (IL)-10 [13], which is also found in solid tumors in vivo [14, 15]. In addition, cell–cell interactions in tumor spheroids constitute a permeable barrier, which is one of the physical factors in TME and makes penetration of CAR T cells through spheroids difficult [15]. Hence, the cytotoxicity of CAR T cells against solid tumors is better simulated using tumor spheroids than using two-dimensional (2D) cultured cells.

Conventional methods used to form spheroids include cell suspension culture [16], non-adherent surface [17, 18], and hanging drop method [19]. These methods are laborious, cumbersome and often generate spheroids of varying sizes [19, 20]. However, the recent use of micro-molding [21], photolithography [22] and 3D bioprinting [23] has shown promise in overcoming these problems. In particular, Zhao et al. reported a method of combining a 3D-printed hanging drop array with a standard 96/384-well plate, which could seamlessly perform the subsequent analyses of drug testing and tumor migration [23]. Despite these advantages, their method would require tedious liquid handling steps to separate CAR T cells and tumor cells killed by CAR T cells from spheroids when performing a cytotoxicity assay. In fact, this separation issue also occurs in other methods that use spheroids.

**Fig. 1:**
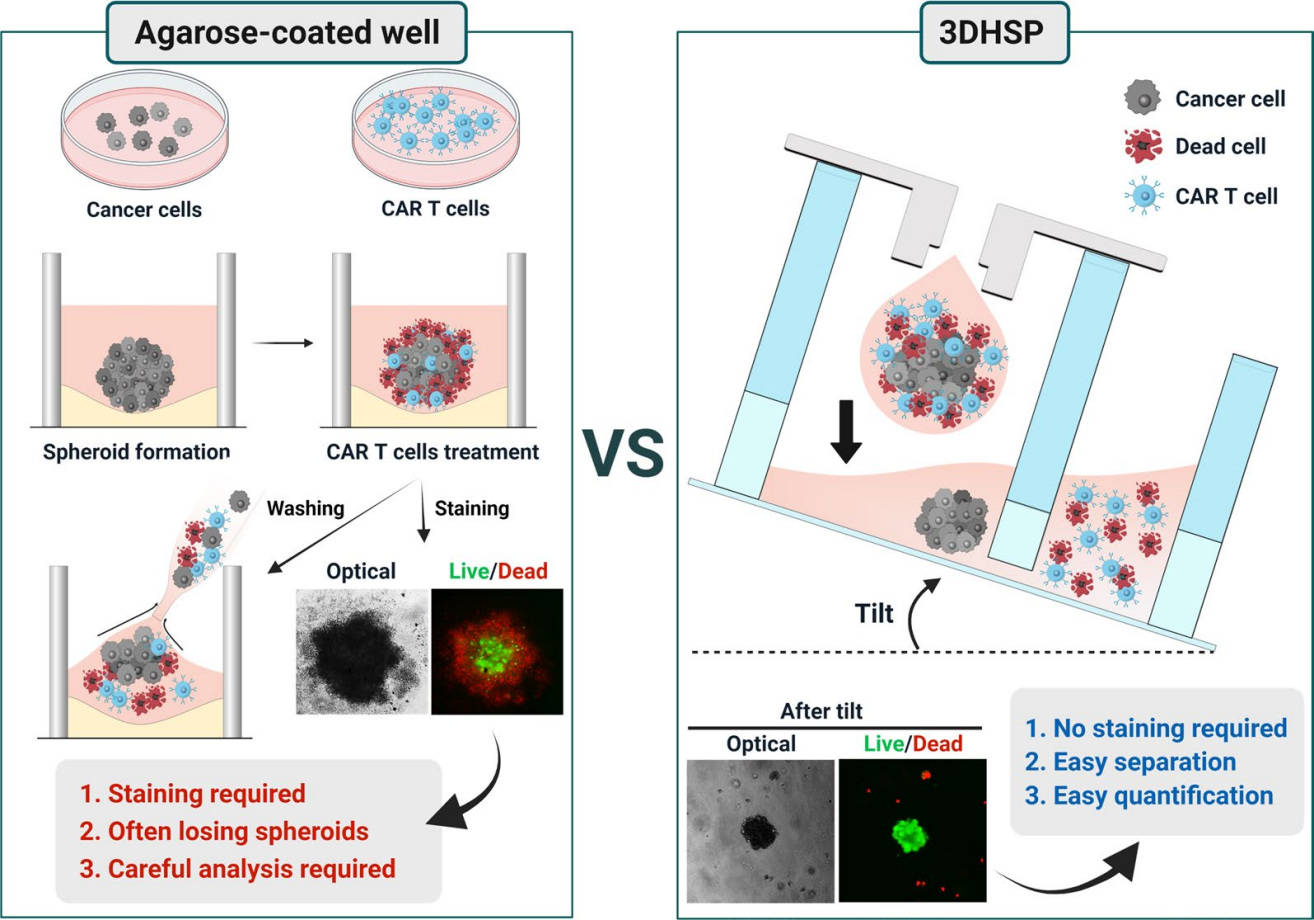
Cytotoxicity assay of chimeric antigen receptor (CAR) T cells in agarose-coated wells. The cytotoxicity was compared to that of the three-dimensional (3D) hanging spheroid plate (3DHSP)

Here, we report a 3D hanging spheroid plate (3DHSP) that enables spheroid formation and separation of unbound and dead T and tumor cells from spheroids during cytotoxicity assays (Fig. 1). To demonstrate the advantages of the 3DHSP over conventional spheroid culture-based CAR T cytotoxicity assays using agarose-coated wells, human epidermal growth factor receptor 2 (HER2)-CAR T cells were tested for their capacity to kill HER2-positive breast tumor cell lines (BT474 and SKOV3) and a HER2-negative glioma cell line (U87) in the 3DHSP. Unlike agarose-coated wells, the 3DHSP did not require live/dead staining for the assay because unbound and dead CAR T and tumor cells were easily separated from spheroids by tilting the 3DHSP at 20–40°. By measuring the size of spheroids in the 3DHSP using optical imaging, the cytotoxic effects of CAR T cells on the spheroids can be assessed. The results suggest that the 3DHSP could be integrated into an HTS system to screen for CARs that enable T cells to kill spheroids formed from a specific tumor type with high efficacy or for spheroids consisting of tumor types that can be killed efficiently by T cells bearing a specific CAR.

## Results and discussion

### Design of 3DHSP

**Fig. 2:**
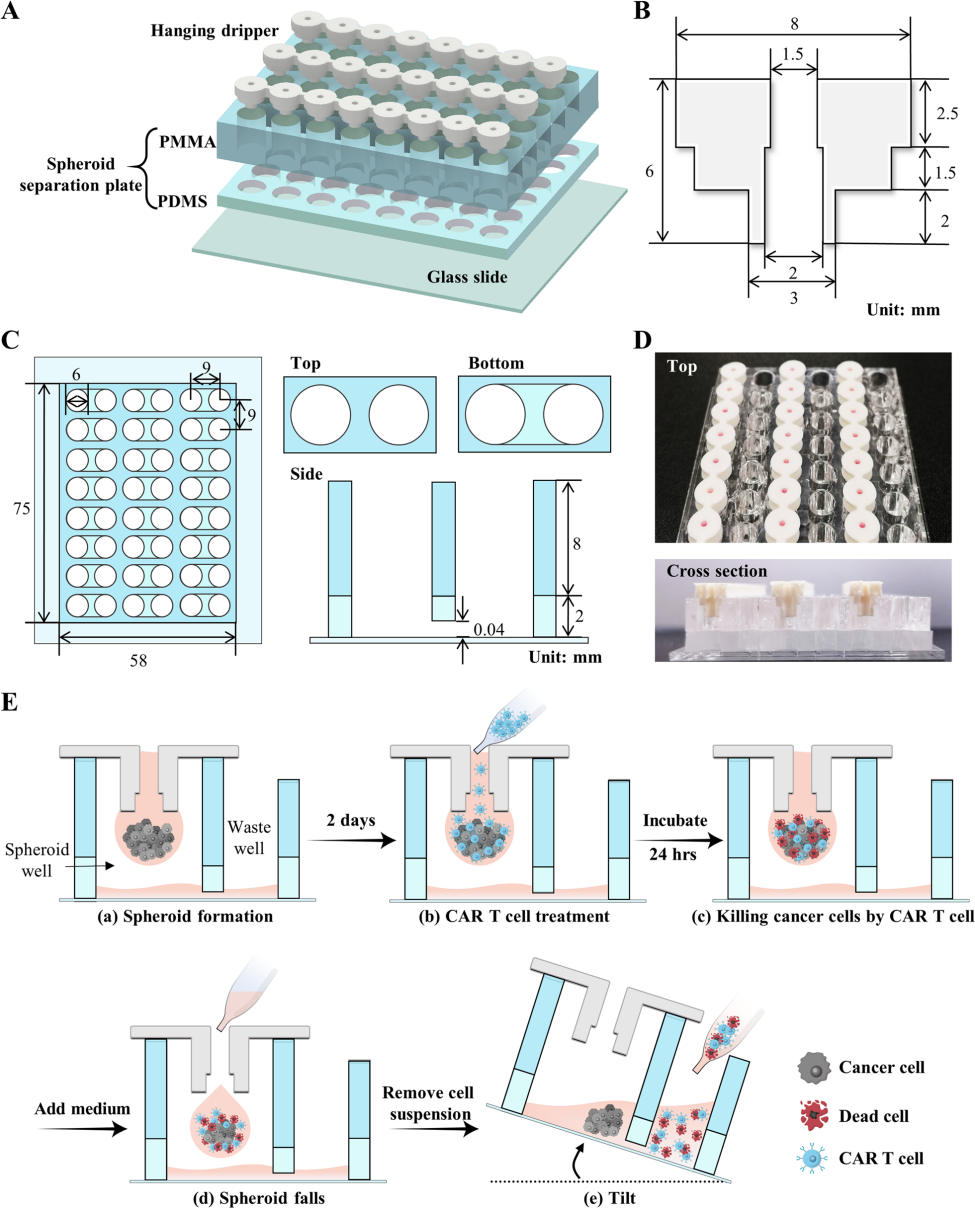
Design and fabrication of 3DHSP. **A** Components of the 3DHSP, a hanging dripper, and a spheroid separation plate. The hanging dripper was 3D-printed, while the separation plate was fabricated by the assembly of poly (methyl methacrylate) (PMMA) and polydimethylsiloxane (PDMS) layers with hole structures. Structures and dimensions of the hanging dripper (**B**) showing its side view and the spheroid separation plate (**C**) showing the top, bottom, and side views. **D** Real image of the 3DHSP. **E** Schematic describing tumor spheroid formation and CAR T assay in a 3DHSP

The 3DHSP is a 24-well plate, and each well is composed of a hanging dripper, spheroid wells and waste wells (Fig. 2 A–D). Each hanging dripper (8 mm and 6 mm in diameter and height, respectively) contains a center hole (3 mm and 2 mm in outer and inner diameters, respectively) (Fig. 2B). The dimensions of the spheroid separation plate are 75 mm (L) ⋅ 58 mm (W) × 10 mm (H) (Fig. 2C), and the plate contains 24 pairs of twin wells interconnected through a channel (6 mm (W) × 40 ${\upmu }\text{m}$(H)). The cytotoxicity assay of CAR T cells to tumor spheroids was performed in the 3DHSP using the following steps: tumor cells were suspended in the hanging dripper and incubated to form tumor spheroids; CAR T cells were added into the hanging dripper to treat the tumor spheroids. The medium was added into the hanging drop so that the tumor spheroid dripped into the spheroid well; dead cells and unbound CAR T cells were transferred from the spheroid well to the waste well by tilting the whole plate at 20–40°, thereby leaving the tumor spheroid in the spheroid well (Fig. 2E).

### Cytotoxicity assay of HER2-CAR T cells in agarose-coated wells

T cells were stimulated with anti-CD3 and anti-CD28 monoclonal antibodies (mAbs) in the presence of IL-2 for 2 d and transduced with HER2-CAR (Additional file 1: Fig. S1A) retrovirus, as described in “Methods” section. The frequency of HER2-CAR T cells, as assessed using flow cytometry 5 d after transduction, was between 50% and 85% (Additional file 1: Fig. S1B).

**Fig. 3:**
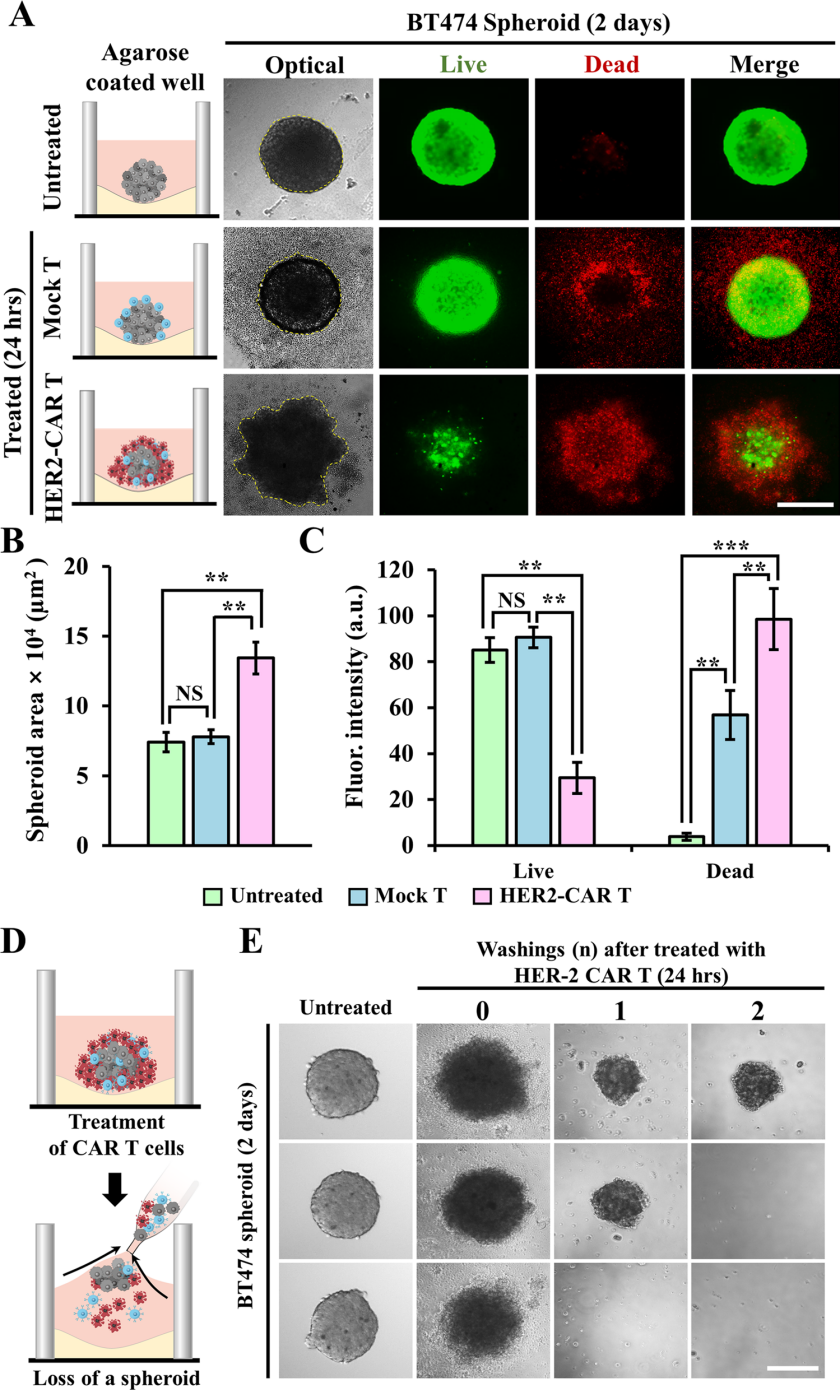
Treatment of BT474 spheroids. BT474 spheroid were treated with either the mock or human epidermal growth factor receptor 2 (HER2)-CAR T cells at a ratio of 1:4 in agarose-coated wells for 24 h. **A** Optical and fluorescent images of the untreated and treated spheroids stained with calcein-AM (green) and ethidium homodimer-1 (red). Scale bar: 200 μm. Area size (**B**) and fluorescence intensity (**C**) of the untreated and treated spheroids. Data are expressed as the mean ± standard error of the mean (SEM) (n = 8). Student’s t-test: **p < 0.01, ***p < 0.001; NS: not significant. **D** Schematic describing the washing steps in agarose-coated wells after BT474 spheroids were treated with HER2-CAR T cells for 24 h. **E** Images of the untreated and treated spheroids with and without one or two repeated washings. Scale bar: 200 μm

The cytotoxic effect of HER2-CAR T cells on spheroids was evaluated by measuring the spheroid size and analyzing the ratios of live and dead cells in the spheroids. The untreated spheroids in the wells were viable while maintaining their round shape, as shown in Fig. 3A. Treatment with mock T cells did not affect the spheroid size or cell viability (Fig. 3A–C). However, there was a significant increase in red fluorescence intensity in the treated spheroids compared to that in the untreated spheroids (Fig. 3A and C), partly owing to the settlement of dead mock T cells onto the spheroids (Additional file 1: Fig. S2). In addition, dead tumor cells might contribute to an increase in fluorescence intensity. This implies that a washing step for the removal of dead cells from agarose-coated wells is required for the cytotoxicity assay to obtain accurate quantification.

The size of spheroids treated with HER2-CAR T cells was significantly larger than those treated with either untreated or mock T cells (p < 0.01) (Fig. 3B). This increase could be due to the swelling of spheroids by the attack of HER2-CAR T cells, similar to the observations made by Szöőr et al. [24]. Despite the increase in size, only live cells remained in the core of the spheroids, whereas dead cells were found at the periphery of the spheroid core (Fig. 3A). This resulted in low and high fluorescence intensities of calcein AM and ethidium homodimer-1 (EthD-1), respectively (Fig. 3C). These results showed that HER2-CAR T cells killed BT474 cells and penetrated the spheroids [25].

Although the cytotoxic effect of CAR T cells on tumor cells can be clearly observed, staining-based methods are not particularly suitable for high-throughput cytotoxicity assays. In general, fluorescent staining of cells with a dye takes at least 30 min, followed by fluorescent imaging and analysis steps [26]. To increase the speed of the cytotoxicity assay, the size of spheroids needs to be directly measured, without staining. However, direct measurement was hampered by the presence of dead cells, including dead HER2-CAR T cells, near the spheroids (Fig. 3A). For example, HER2-CAR T cells were bound to the spheroids and increased the size of the spheroids (Fig. 3A and B), which is contradictory to the observation of fluorescent staining results (Fig. 3C). This suggests that for direct measurement of spheroid size after CAR T cell treatment, removal of dead CAR T cells and tumor cell-form spheroids is necessary. However, it was difficult to remove dead cells from the spheroids. Washing resulted in the loss of spheroids [27] in the agarose-coated wells (Fig. 3D). In fact, repeated washings removed most of the spheroids from the wells. This technical challenge led to the development of a hanging drop method that can easily remove unbound and dead cells from spheroids after CAR T cell treatment.

### Characterization of spheroid formation in 3DHSP

**Fig. 4:**
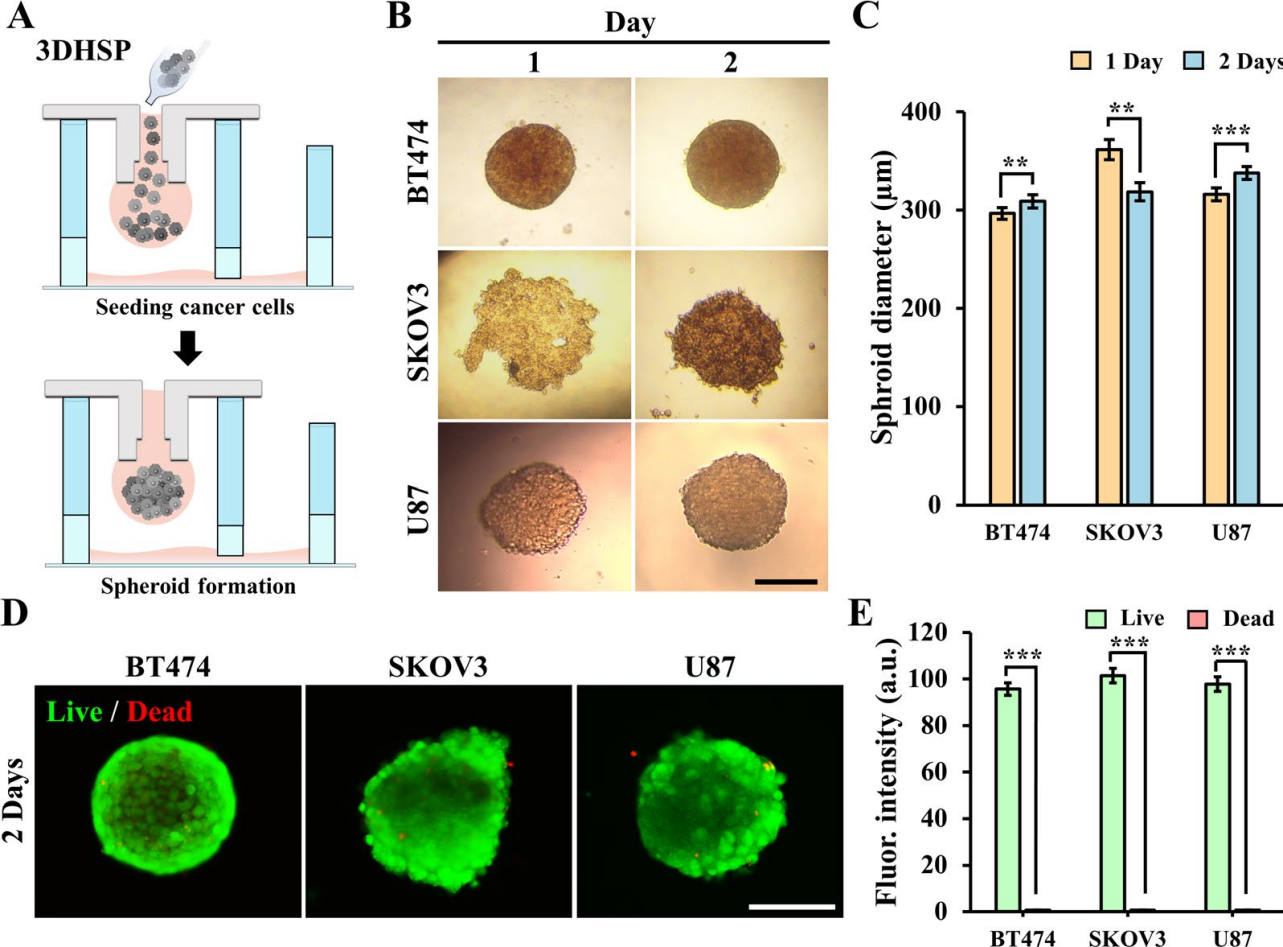
Characterization of tumor spheroids generation in the 3DHSP. **A** Schematic describing spheroid generation in the 3DHSP. **B** Optical images of BT474, SKOV3, and U87 spheroids in the 3DHSP for 1 and 2 d. Scale bar: 200 μm. **C** Size of the spheroids after 1 and 2 d. **D** Representative fluorescent images of three kinds of tumor spheroids for 2 d, showing the live (green) and dead (red) cells. **E** Fluorescence intensity of live/dead cells in three types of tumor spheroids for 2 d (n ≥ 10). Student’s t-test: **p < 0.01, ***p < 0.001. Scale bar: 200 μm

About 3000 tumor cells were cultured in the 3DHSP for one or two days to form spheroids (Fig. 4A). After 1 d, BT474 and U87 cells formed round tumor spheroids, whereas SKOV3 cells formed sheet-like cell aggregates. These differences in spheroid formation may be due to variations in the cell aggregation capacity across cell lines [28] (Fig. 4B). SKOV3 cells seemed to require a longer time to form a tighter spheroid than U87 and BT474 cells. After 2 d, unlike the BT474 and U87 spheroids, the SKOV3 aggregates became tighter, resulting in a reduction in their size (Fig. 4C). After 2 d, the size of spheroids formed by all the three cell lines was close to 300 μm: BT474, 297.1 ± 6.7 μm; U87, 335.4 ± 7.1 μm; SKOV3, 314.2 ± 15.9 μm (Fig. 4C). The viability of the three types of tumors remained high (green fluorescence intensity), while the number of dead cells was almost negligible (red fluorescence intensity; Fig. 4D and E). The results showed that the 3DHSP was very effective in forming spheroids of similar sizes without compromising cell viability. Furthermore, we verified the effects of cell number on spheroid formation and the long-term viability of cells in the 3DHSP. To check the effect of cell number on spheroid formation, BT474 cells were added to the 3DHSP at 300, 3000 and 30,000 cells suspended in 25 µL culture media. The results showed that the cells formed uniform tumor spheroids at all cell numbers, even at lower cell numbers (300 cells). At all cell concentrations, the tumor spheroids maintained high cell viability after 7 d of culture (Additional file 1: Fig. S3).

### Effect of 3DHSP tilt angle on spheroid size

**Fig. 5:**
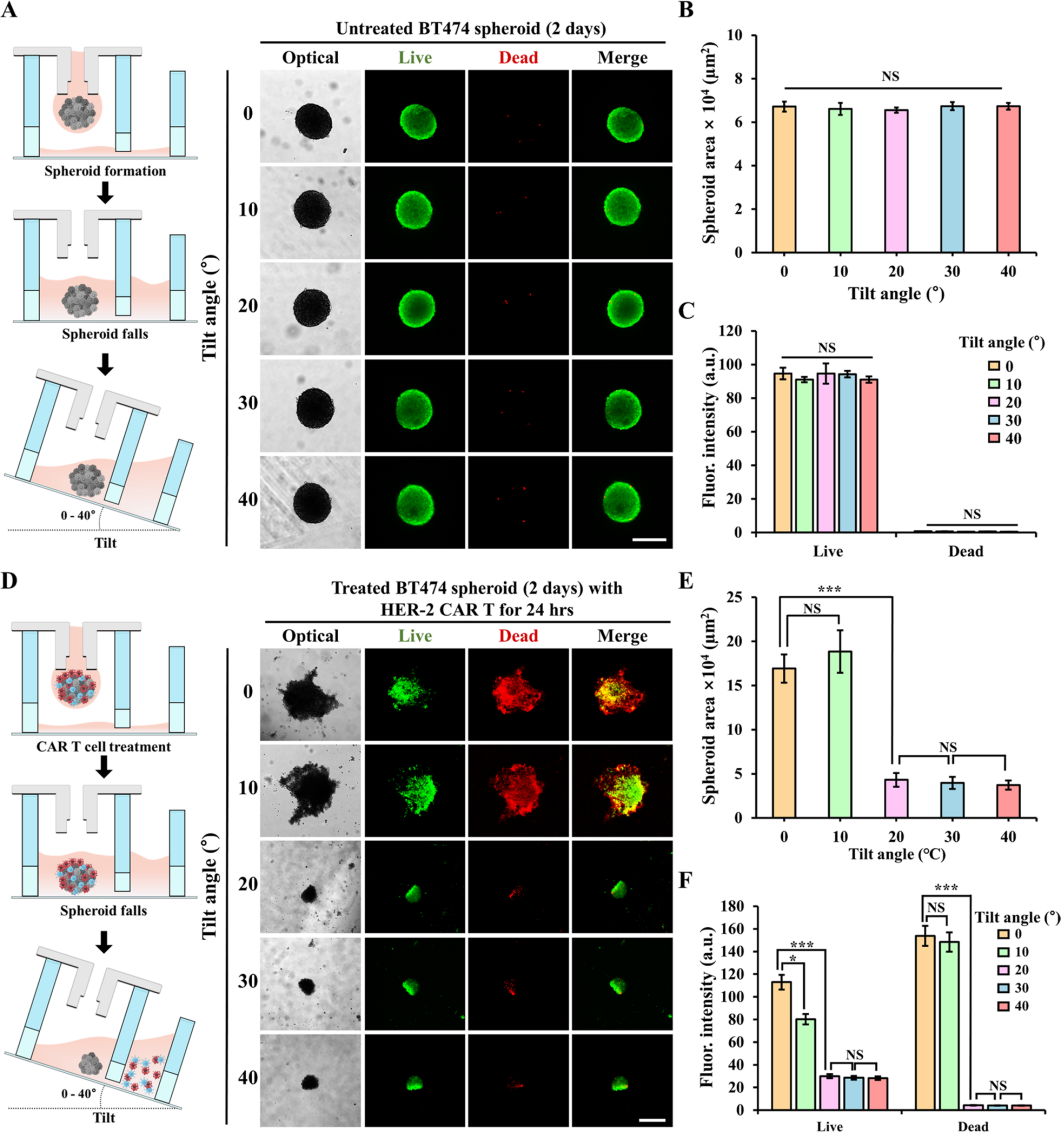
Effect of 3DHSP tilt angle on the separation of remaining tumors. **A** Optical and fluorescent images of untreated spheroids stained with calcein-AM (green) and ethidium homodimer-1 (red) at 3DHSP tilt angles of 0°, 10°, 20°, 30°, and 40°. Scale bar: 300 μm. **B** Areas of BT474 spheroids at 3DHSP tilt angles of 0°, 10°, 20°, 30°, and 40°. **C** Fluorescence intensity of live/dead cells in tumor spheroids at different tilt angles. **D** Optical and fluorescent images of HER2-CAR-T cell-treated spheroids stained with calcein-AM and ethidium homodimer-1 at 3DHSP tilt angles of 0°, 10°, 20°, 30°, and 40°. Scale bar: 200 μm. **E** Areas of BT474 spheroids at 3DHSP tilt angles of 0°, 10°, 20°, 30°, and 40°. **F** Fluorescence intensity of live/dead cells in tumor spheroids at 3DHSP tilt angles of 0°, 10°, 20°, 30°, and 40°. Student’s t-test: *p < 0.05, ***p < 0.001; NS: not significant

To verify that the 3DHSP tilting process did not affect the size of the spheroids, BT474 spheroids without CAR T treatment were cultured in hanging drops for 2 d, after which they were deposited into the spheroid separation well. The size of the spheroids and fluorescence intensity were measured at tilt angles of 0°, 10°, 20°, 30°, and 40° (Fig. 5A–C). The results show that the size of the spheroids and the fluorescence intensity of dead and live cells were not significantly affected by the different tilt angles (Fig. 5B and C). After the spheroids were treated with CAR T cells, dead tumor cells and CAR T cells were loosely attached to the spheroids (Additional file 2: Movie S1). To verify the effect of the tilt angle of the 3DHSP on the separation of surviving tumors, BT474 spheroids were treated with HER2-CAR T cells for 24 h in the 3DHSP. The size of ​​cell aggregates was measured at tilt angles of 0°, 10°, 20°, 30°, and 40° (Fig. 5D–F). Owing to loosely associated dead cells to the aggregates, at a 10° tilt, the size of ​​the cell aggregates seemed to be larger than that at a 0° tilt. As the tilt angle increased, the flow rate increased, and the cells around the tumor spheroids were washed away. Therefore, there was a significant reduction in spheroid size at a tilt angle of 20° (Additional file 3: Movie S2). Compared with the spheroid size at 20° tilt angle, the sizes of ​​the spheroids at 30° and 40° tilt angles were similar (Fig. 5E). Live/dead staining further verified the separation capability of the platform. It was shown that almost all dead cells were removed by the washing step from the trapped spheroids in the spheroid well, and only spheroids were left at tilt angles of 20°, 30°, and 40°. At tilt angles of 0° and 10°, there was still a mixture of live and dead cells in the spheroid well. The fluorescence intensity of live cells at tilt angles of 20°, 30°, and 40° were much lower than those at tilt angles of 0° and 10°, which indicates that the surviving CAR T cells were washed away. At tilt angles of 20°, 30°, and 40°, the green fluorescence intensity in the spheroids was approximately the same, which means that the trapped spheroids had stabilized, and the surrounding cells (dead cells and CAR T cells) were removed by washing. The reason for the increase in red fluorescence intensity, compared with that in the 0° tilt angle, is the slight spread of loosely bound dead cells at a tilt angle of 10° (Fig. 5F). However, owing to the increased flow rate above a tilt of 20°, these loosely bound cells dissociated and flushed away into the waste well. At tilt angles lower than 20°, only a small number of dead cells in the spheroid well were flowed to the waste well, making it difficult to separate dead cells from spheroids. At tilt angles higher than 40°, spheroids were stuck in the flow channel, causing media to overflow over the spheroid well. Therefore, the operation of the 3DHSP should be limited to a tilt of 20–40°. Live/dead staining showed that only less than 8% dead cells remained in the spheroids after the tilting, indicating that the sensitivity of the detachment was estimated to be greater than 92%. In addition, the results showed that the spheroids were not lost by washing when the diameter of the remaining tumor spheroids was greater than 40 μm (Additional file 1: Fig. S4).

### Cytotoxicity of HER2-CAR T cells in 3DHSP

**Fig. 6:**
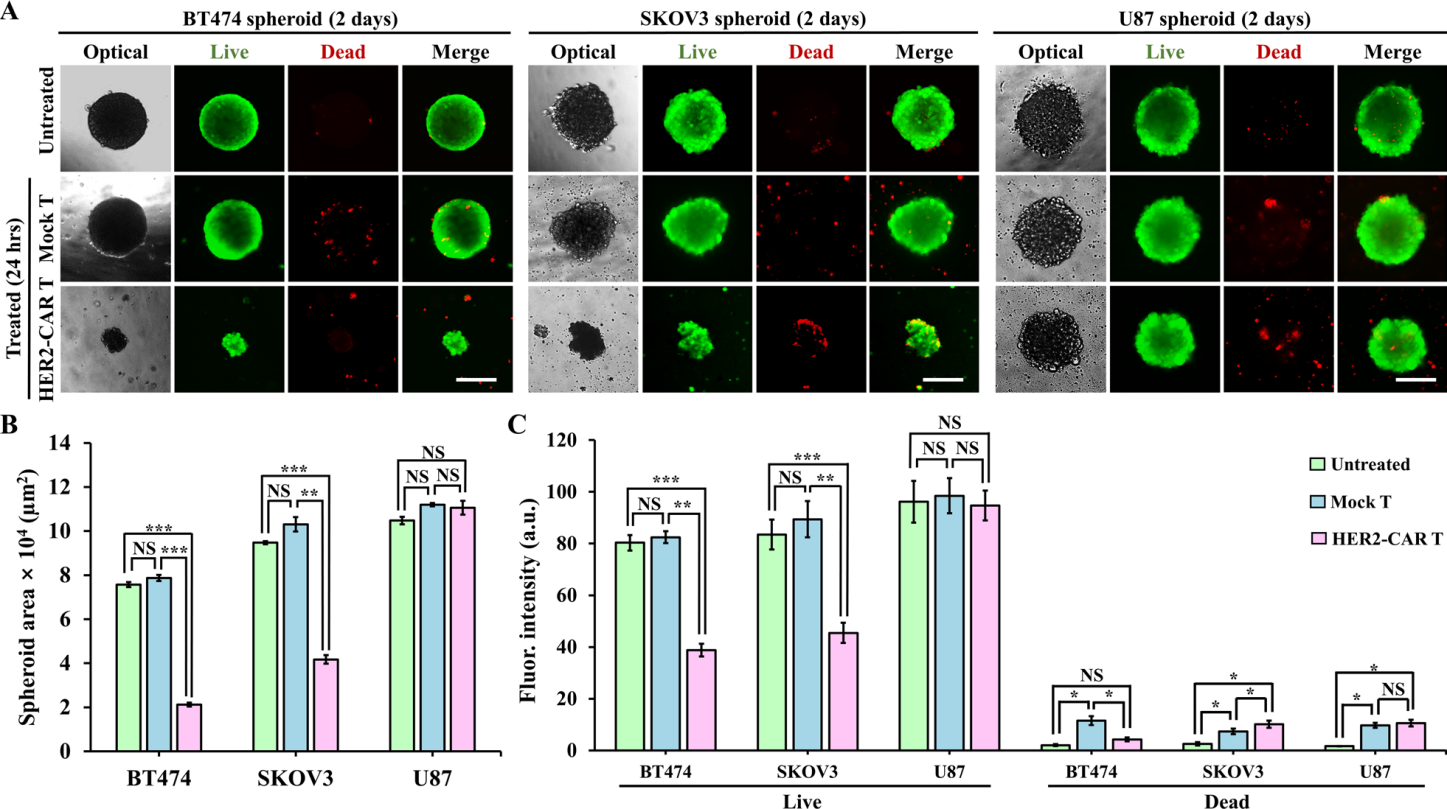
Cytotoxicity assay of the mock T and HER2-CAR T cells in spheroids in the 3DHSP. **A** Optical and fluorescent images of the untreated and treated spheroids of BT474, SKOV3, and U87 cells stained with calcein-AM (green) and ethidium homodimer-1 (red). Scale bar: 200 μm. **B** Area and green and red fluorescence intensity (**C**) of the untreated and treated spheroids. Student’s t-test: *p < 0.05, **p < 0.01, ***p < 0.001; NS: not significant

Mock T and HER2-CAR T cells were used to verify the cytotoxic effects on BT474, SKOV3, and U87 spheroids in the 3DHSP. In the case of BT474 spheroids treated with mock T cells, the diameter of the spheroids was the same as that of the untreated spheroids. Live/dead staining showed that most of the tumor cells in the spheroids were viable (Fig. 6A), with hardly any dead tumor cells or CAR T cells. In comparison, in the case of BT474 spheroids treated with HER2-CAR T cells, the size of the spheroids decreased. At the effector to target ratio (4:1), HER2-CAR T cells invaded BT474 spheroids, destroying all the tumor spheroids with only a small aggregate of cells remaining. Similar cytotoxic effects were observed in SKOV3 spheroids [29]. At the effector to target ratio (1:1), HER2-CAR T cells invaded BT474 and SKOV3 spheroids, but only the cells in the outer layer of the spheroids were destroyed, and a large aggregate of tumor cells remained (Fig. S5). For HER2-negative U87 spheroids, HER2-CAR T and Mock T cells showed no cytotoxic effects at 1:1 an 4:1 of effector to target ratios [30]. Green fluorescence intensity was compared to the size of the BT474 spheroid, which decreased in HER2-expressing tumor cells (Fig. 6B and C). This suggests that in the 3DHSP, the cytotoxicity of CAR T to tumor spheroids could be measured without any staining. The lack of a red fluorescence signal suggests that dead cells were washed away (Fig. 6C). This shows that all cells apart from the spheroids can be removed from the spheroid well and that the size of the trapped spheroids can be used to measure the cytotoxic effect of HER2-CAR T cells.

The key step of the platform was to co-culture HER2-CAR T cells with pre-formed tumor spheroids in situ and then wash out all cells except the spheroids for image-based quantitative analysis of the HER2-CAR T cytotoxicity. It is noteworthy that this platform eliminates the need for cumbersome transport and washing procedures, which are usually inevitable in downstream cell spheroid analysis [31], as washing and staining after co-culture of immune cells and tumor spheroids are usually required. Unlike other devices, 3DHSP does not require both expensive fluorescent staining and washing steps for spheroid size measurement [2, 24, 32] (Table 1). In addition, this platform enables real-time visualization of HER2-CAR T cells infiltrating into the tumor spheroid.

**Table 1:** Advantages of the 3DHSP over other devices in screening of effects of immune cytotoxic cells on tumor spheroids

Methods	Ultra-low attachment 96-well round bottom plate [2, 24, 32]	Agarose-coated well	3DHSP
Spheroid morphology	Uniform	Uniform	Uniform
Easiness in separating dead cells from spheroids	Cumbersome (pipetting)	Cumbersome (pipetting)	Easy
Loss of spheroids	Often	Often	Rare
Live/dead staining	Required	Required	Not required
Screening of effect of anti-cancer drugs or immune cytotoxic cells on tumor spheroids	Difficult	Difficult	Easy

### Cytotoxicity of HER2-CAR T cells in 2D culture

To further validate the cytotoxicity results for the 3DHSP, a 2D cytotoxicity assay was carried out for comparison. Mock or HER2-CAR T cells were co-cultured with the same tumor cells used in the 3DHSP but engineered to express luciferase at the same effector to target ratio of 4:1 in 2D culture using 96-well plates. As shown in Additional file 1: Fig. S6, Mock T cells expectedly showed negligible killing of all tumor cells such that their viable numbers were comparable to those without CAR T cells (untreated). HER2-CAR T cells mediated substantial lysis of HER2-expressing SKOV3 cells but not U87 cells that lack HER2 expression. In contrast to results obtained with 3DHSP, HER2-CAR T cells did not kill HER2-expressing BT474 cells in 2D culture. We observed that while SKOV3 cells spread their bodies more uniformly across the well surface, BT474 cells formed compact clusters and spread their cell bodies to varying extents over the well surface. The non-uniform confluency of BT474 cells may explain their lower susceptibility compared with SKOV3 cells to killing by HER2-CAR T cells. Only when the effector to target ratio was raised to 16:1 did HER2-CAR T cells show lysis of BT474 cells. Collectively, our findings argue for an important advantage the 3DHSP system holds over 2D co-culture in that the former enables uniform and consistent distribution of tumor cells in spheroids, independent of tumor type, to reduce the likelihood of variability in growth of tumor cells which occurs in 2D culture from confounding the assessment of CAR T-mediated anti-tumor cytotoxicity.

## Conclusions

In this study, we described the 3DHSP that enables the formation of tumor spheroids and separation of unbound and dead cells from the spheroids. By tilting the 3DHSP, we overcame the difficulty of separating unbound CAR T cells and dead tumor cells from the spheroids, which is a major problem when using tumor spheroids for CAR T cells in standard cytotoxicity assays using agarose wells. Thus, the 3DHSP does not require cumbersome transport and washing procedures, which are usually inevitable in downstream cell spheroid analysis. The results show that with our system, the size of the spheroid can be optically determined without the use of any fluorescence tags, such as live/dead staining. Owing to its simplicity of operation, we propose that the 3DHSP can be incorporated into a HTS system to screen for CARs that enable T cells to kill spheroids formed from a selected tumor type with high efficacy or for spheroids made from diverse tumor cell types that are susceptible to killing by T cells bearing a selected CAR. Our microfluidic platform can also aid in screening the effects of CAR-natural killer (NK) cells and anti-cancer drugs on tumor spheroids.

## Methods

### Primary cells and cell lines

Frozen human peripheral blood mononuclear cells (PBMCs) from various de-identified donors (STEMCELL Technologies, Vancouver, BC, Canada) were thawed, washed, and suspended in the Roswell Park Memorial Institute (RPMI)-1640 (HyClone Laboratories, Logan, UT, USA) complete medium containing 10% (v/v) fetal bovine serum (FBS; HyClone), 100 units/mL of penicillin (Life Technologies, Carlsbad, CA, USA), and 100 mg/mL of streptomycin (Life Technologies) to prepare for subsequent cultures.

Phoenix-GP cells stably expressing MoMLV gag-pol (American Type Culture Collection (ATCC) #CRL-3215; Bethesda, MD, USA) and PG13 cells (ATCC #CRL-10686) were maintained in Dulbecco’s modified Eagle’s medium (DMEM) (#08458-45; Nacalai Tesque, Kyoto, Japan) supplemented with 10% (v/v) FBS.

Luciferase-expressing cell lines, BT474 (breast ductal carcinoma) (a kind gift from Dr. Jeong Eon Lee from Samsung Medical Center) and SKOV3 (ovarian adenocarcinoma, #1594), were provided by the Japanese Collection of Research Bioresources (JCRB, Osaka, Japan) Cell Bank. U87 glioma cells purchased from ATCC were engineered to express luciferase by lentiviral transduction with pLenti CMV Puro LUC (Addgene #17477; Watertown, MA, USA) and treated with puromycin (InvivoGen, San Diego, CA, USA) for at least 1 week. Subsequently, a single cell clone of U87 with high luciferase expression was obtained by limiting dilution. Parental U87 cells were maintained in Minimum Essential Media (MEM; Life Technologies), while luciferase-expressing U87, BT474, and SKOV3 cells were maintained in RPMI-1640 supplemented as above. All the cells were cultured at 37 °C and 5% carbon dioxide (CO_2_).

### HER2-CAR vector construction and generation of CAR T cells

The HER2-CAR vector consists of a HER2-binding single-chain variable fragment (scFv) moiety bearing a 4D5 sequence derived from the humanized monoclonal antibody trastuzumab (trastuzumab) [33], a CD8a hinge and transmembrane region, and intracellular domains of CD3z and CD28 co-stimulatory receptors. Sequences were synthesized (Bio Basic, Markham, Ontario, Canada) and sub-cloned into the MSGV Hu Acceptor retroviral vector (Addgene #64269) after removal of human T-cell receptor (TCR) genes.

Phoenix-GP cells were co-transfected with the HER2-CAR vector and pCMV-VSV-G (Addgene #8454) using FuGENE 6 (Promega, Madison, WI, USA). CAR retroviral supernatant was collected 24–48 h post-transfection, filtered with a 0.45 μm filter, and applied in the presence of 8 mg/mL polybrene to PG13 cells by spinoculation at 600×*g* for 2 h to generate cells producing GaLV-pseudotyped HER2-CAR retrovirus (HER2-CAR PG13 cells), which were expanded and cryopreserved. One week before T cell activation, HER2-CAR and parental PG13 cells were thawed and cultured to generate HER2-CAR and mock viral supernatants. Non-tissue culture-treated plates were pre-coated with RetroNectin (rFN-CH-296, #T100B; Takara Bio Inc., Otsu, Japan) at 5.26 µg/cm^2^ as per the manufacturer’s instructions. The viral supernatant was filtered and added to RetroNectin-coated wells, followed by centrifugation at 1500×*g* for 2 h. The cells were activated with 50 ng/mL soluble anti-CD3 (OKT3; eBioScience, San Diego, CA, USA) and 100 ng/mL soluble anti-CD28 (CD28.2; eBioScience) monoclonal antibodies (mAbs) in the presence of 20 IU/mL recombinant human interleukin-2 (IL-2; Peprotech, East Windsor, NJ, USA) for 2 d and then transduced with HER2-CAR virus-bound or unbound RetroNectin at 600×*g* for 30 min to generate HER2-CAR and mock T cells, respectively. Transduced T cells were expanded in the presence of 100 IU/mL IL-2 for 5–7 d before cryopreservation. HER2-CAR and mock T cells were thawed and rested overnight in complete medium prior to co-culture with 2D tumor cell culture or spheroids.

### Flow cytometry

Cells were first treated with Human TruStain FcX (Fc receptor blocking solution; #422302; BioLegend) and then incubated with recombinant biotinylated protein L (#RPL-P814R; ACROBiosystems, Beijing, China) followed by PE-conjugated streptavidin (#12-4317-87; eBioscience) to assess HER2-CAR expression. Then, 4’,6-diamidino-2-phenylindole dihydrochloride (DAPI; Sigma-Aldrich, St. Louis, MO, USA) was used to exclude the dead cells. Samples were acquired on a MACSQuant X cytometer (Miltenyi Biotec, Auburn, CA, USA) and analyzed using the FlowJo software (TreeStar Inc., Ashland, OR, USA).

### Cytotoxicity assay of the mock and HER2-CAR T cells in 2D culture

Mock or HER2-CAR T cells were co-cultured with 1 × 10^4^ luciferase-expressing tumor cells at an effector (CAR T) to target (tumor) ratio of 4:1 in 96-well plates for 24 h in triplicate. Surviving tumor cells were assessed for luciferase activity using the Bright-Glo Luciferase Assay System (#E2620; Promega) according to the manufacturer’s protocol, with the luciferase reagent diluted with phosphate-buffered saline (PBS, pH 7.4) at a 1:1 ratio prior to use. Cell culture media were removed from the wells, and 100 µL of the diluted luciferase reagent was added to each well. The plates were shaken for 5 min to allow complete lysis of the cells before measurement. Luminescence of the lysed mixture was measured using the Synergy HTX Multi-Mode Microplate Reader (BioTek Instruments, Winooski, VT, USA).

### Spheroid formation in agarose-coated wells

Agarose-coated wells were prepared by adding 50 µL of 1.5% (w/v) agarose (Sigma-Aldrich) at 70 °C in distilled water into 96-well plates (Corning Inc., Corning, NY, USA) and solidifying the agarose at 25 °C room temperature in a biosafety cabinet according to a previously described method [9, 10]. A tumor cell suspension was prepared in RPMI-1640 at a density of 20,000 cells/mL, and 150 µL of the suspension was poured into each well. Then, the cells in the well were incubated at 37 °C with 5% CO_2_ for 48 h until they formed a spheroid with a diameter of approximately 300 μm.

When approximately 3,000 cells (BT474) were incubated in the agarose-coated wells, they started to form a single spheroid at 24 h and became larger up to approximately 300 μm in diameter after 48 h.

### Cytotoxicity assay of the mock and HER2-CAR T cells in agarose-coated wells

A spheroid in each well was mixed with 10 µL of RPMI-1640 containing either 12,000 mock or HER2-CAR T cells. A single spheroid in each well was then mixed with either mock T or HER2-CAR T cells at an effector to target ratio of 4:1 for 24 h. The wells were incubated at 37 °C in 5% CO_2_ for 24 h. Then, 10 µL of LIVE/DEAD® Viability/Cytotoxicity Kit reagent (Molecular Probes, Eugene, OR, USA) was added to the wells and incubated at 37 °C with 5% CO_2_ for 30 min. Optical and fluorescent images were captured using a fluorescent microscope (DeltaVision Elite; GE Healthcare, Chicago, IL, USA). Images were processed and analyzed using the ImageJ software (NIH, Bethesda, MD, USA).

### Fabrication of 3DHSP

The hanging dripper was designed using Inventor 2020 (Autodesk, San Rafael, CA, USA) and printed using a 3D printer (Cubicon, Seongnam, Korea) with acrylonitrile butadiene styrene (ABS) filaments. The plate consisted of two layers, of which the upper layer was made of poly (methyl methacrylate) (PMMA) (ENGP, Incheon, Korea), and the lower layer was made of polydimethylsiloxane (PDMS) (Dow Corning Co., Midland, MI, USA) [34]. A PMMA sheet with a thickness of 8 mm was ablated into a layer of 75 mm (L) × 58 mm (W) and holes with a diameter of 6 mm using a laser cutter (Zing™ 24 Laser; Epilog Laser, Golden, CO, USA). A PDMS layer of 2 mm thickness was fabricated by soft lithography using a silicon mold [34] and holes were punctured into the layer using a 6 mm biopsy punch (Kai Industries Co., Gifu, Japan). Both the PDMS layer and glass slide were treated with oxygen plasma for 30 s and then bound to each other. Subsequently, the PMMA layer was pasted with a mixture of PDMS and the curing agent (10:1 ratio) (w/w) on the bottom side and then placed onto the PDMS layer at 80 °C for 2 h until both layers were bounded.

### Spheroid formation in 3DHSP

Before seeding tumor cells, the 3DHSP cells were thoroughly washed with 70% ethanol, rinsed twice with PBS, and irradiated with ultraviolet light for 30 min, detached from a Petri dish using trypsin/ethylenediaminetetraacetic acid (EDTA) solution (Sigma-Aldrich), and then neutralized with RPMI-1640.

A cell suspension was prepared in RPMI-1640 at a density of 120,000 cells/mL. Approximately, 3000 cells in 25 µL of RPMI-1640 were seeded into the 3DHSP through a hanging dripper using a pipette. Parafilm (Heathrow Scientific, Vernon Hills, IL, USA) was used to wrap the 3DHSP to prevent evaporation of the medium. The 3DHSP were incubated at 37 °C and 5% CO_2_ for 48 h until the cells formed a spheroid with a diameter of 300 μm in each hanging drop, while fresh RPMI-1640 was added to it by removing 7 µL of media from the 3DHSP through the dripper and adding 10 µL of fresh RPMI-1640 into the dripper [23].

### Cytotoxicity assay of the mock and HER2-CAR T cells in 3DHSP

The 3DHSP assay was similarly performed as in agarose-coated cells (Fig. 4E). Each spheroid with a diameter of approximately 300 μm in the well was mixed with 10 µL of RPMI-1640 containing 12,000 either mock or HER2-CAR T cells. The 3DHSP was incubated at 37 °C in 5% CO_2_ for 24 h and then fixed on a tilting mechanical stage (Thorlabs, Newton, NJ, USA) with a custom-built computer program Kinesis® (Thorlabs) (Additional file 4: Movie S3). A spheroid in the hanging dripper was deposited into the spheroid separation plate by injecting 100 µL of RPMI-1640 into the hanging dripper using a pipette, while dead and detached cells were separated from the spheroid by tilting the 3DHSP by 20°–40°. The remaining spheroid in the well was stained with 10 µL of LIVE/DEAD® Viability/Cytotoxicity Kit reagent as described above. Imaging of spheroids and analysis were performed as described above.

### Rate of loss of spheroids after washing in agarose-coated wells and 3DHSP

To compare the rate of loss of spheroids after washing in agarose-coated wells and the 3DHSP, 3000 BT474 cells were cultured in agarose-coated wells and the 3DHSP for 48 h to form spheroids, which were treated with 12,000 HER2-CAR T cells for 24 h. For the agarose-coated wells, 100 µL of PBS was added to the wells and then gently pipetted twice. After waiting for 30 s to allow the spheroids to settle down, the upper cell suspension was carefully removed. In the 3DHSP, 100 µL of PBS was added to the hanging drop, the 3DHSP was then tilted 30°, and the cell suspension was removed from the waste well.

### Statistical analysis

All data are expressed as the mean ± standard deviation (SD) from three or more independent experiments. Statistical significance was determined using Student’s *t-test*.

## Supplementary Information

**Additional file 1**: **Fig. S1.** Generation of human epidermal growth factor receptor 2 (HER2)-chimeric antigen receptor (CAR) T cells. (A) Schematic diagram showing HER2-CAR structure. (B) Frequency of HER2-CAR T cells as assessed using flow cytometry. Data in (B) are representative of three independent experiments: HTM, hinge and transmembrane region. **Fig. S2.** Treatment of BT474 spheroids with either mock T or HER2-CAR T cells. The ratio of BT474 spheroids to either mock T or HER2-CAR T cells was 1:4 in agarose-coated wells, and the treatment duration was 3, 16, and 24 h. **Fig. S3.** Formation of BT474 spheroids on the 3DHSP. (A) Micrographs of BT474 spheroids with different cell numbers (300, 3000, and 30,000) on the 3DHSP for 1, 3, 5, and 7 d. (B) Spheroid diameter changing with time. (C) Representative images of BT474 spheroids cultured on the 3DHSP for 7 d showing the live (green) and dead (red) cells. (D) Green and red fluorescence intensity of spheroids cultured on the 3DHSP for 7 d. Scale bar: 200 μm. Student’s t-test: *p < 0.05, **p < 0.01, ***p < 0.001; NS, not significant. **Fig. S4**. Image of the treated BT474 spheroids with and without either one or two repeated washings. Scale bar: 200 μm. **Fig. S5**. Treatment of spheroids with either the mock or HER2-CAR T cells. The ratio of the spheroids and either the mock or HER2-CAR T cells was 1:1 in the 3DHSP, and the treatment duration was 24 h. (A) Optical and fluorescent images of the treated spheroids of BT474, SKOV3, and U87 cells stained with calcein-AM (green) and ethidium homodimer-1 (red). (B) Area and green and red fluorescence intensity (C) of the treated spheroids. Scale bar: 200 μm. Student’s t-test: *p < 0.05, ***p < 0.001; NS, not significant. **Fig. S6.** Cytotoxicity of mock T and HER2-CAR T cells against luciferase-expressing cells co-cultured in 2D. The effector (BT474, SKOV3, and U87 cells) to target ratio was 4:1 (unless otherwise stated), and the cytotoxicity was assessed by the luminescence of surviving tumor cells 24 h later. Data are the mean ± standard deviation (SD) of three technical replicates for each condition and representative of one experiment.

**Additional file 2: Movie S1.**Human epidermal growth factor receptor 2 (HER2)-chimeric antigen receptor (CAR) T cells were treated with BT474 spheroids at an effector to target ratio of 4:1 in the three-dimensional hanging spheroid plate (3DHSP) for 24 h.

**Additional file 3: Movie S2. **HER2-CAR T cells were treated with BT474 spheroids at an effector to target ratio of 4:1 in the 3DHSP for 24 h, and then the 3DHSP was tilted at 20° and back to 0°. The movie duration was 20 s with a 1 s interval.

**Additional file 4: Movie S3.** Operation of tilting the 3DHSP.

## Data Availability

Not applicable.

## Abbreviations

3DHSP: Three-dimensional hanging spheroid plate
ABS: Acrylonitrile butadiene styrene
CAR: Chimeric antigen receptor
DAPI: 4′,6-diamidino-2-phenylindole dihydrochloride
DMEM: Dulbecco’s modified Eagle’s medium
EDTA: Ethylenediaminetetraacetic acid
FBS: Fetal bovine serum
FDA: Food and Drug Administration
HER2: Human epidermal growth factor receptor 2
HTS: High-throughput screening
IL: Interleukin
PBMCs: Peripheral blood mononuclear cells
scFv: Single-chain variable fragment
PDMS: Polydimethylsiloxane
PMMA: Poly (methyl methacrylate)
RPMI: Roswell Park Memorial Institute
TCR: T-cell receptor
TME: Tumor microenvironment
EthD-1: Ethidium homodimer-1
SD: Standard deviation
