# Supplementary material for: 3D hanging spheroid plate for high-throughput CAR T cell cytotoxicity assay
Source: J Nanobiotechnology. 2022 Jan 10;20:30. doi: 10.1186/s12951-021-01213-8 (PMC8744335; doi:10.1186/s12951-021-01213-8)
Supplement: Supplementary file 1 — Additional file 1: Fig. S1. Generation of human epidermal growth factor receptor 2 (HER2)-chimeric antigen receptor (CAR) T cells. (A) Schematic diagram showing HER2-CAR structure. (B) Frequency of HER2-CAR T cells as assessed using flow cytometry. Data in (B) are representative of three independent experiments: HTM, hinge and transmembrane region. Fig. S2. Treatment of BT474 spheroids with either mock T or HER2-CAR T cells. The ratio of BT474 spheroids to either mock T or HER2-CAR T cells was 1:4 in agarose-coated wells, and the treatment duration was 3, 16, and 24 h. Fig. S3. Formation of BT474 spheroids on the 3DHSP. (A) Micrographs of BT474 spheroids with different cell numbers (300, 3000, and 30,000) on the 3DHSP for 1, 3, 5, and 7 d. (B) Spheroid diameter changing with time. (C) Representative images of BT474 spheroids cultured on the 3DHSP for 7 d showing the live (green) and dead (red) cells. (D) Green and red fluorescence intensity of spheroids cultured on the 3DHSP for 7 d. Scale bar: 200 μm. Student’s t-test: *p < 0.05, **p < 0.01, ***p < 0.001; NS, not significant. Fig. S4. Image of the treated BT474 spheroids with and without either one or two repeated washings. Scale bar: 200 μm. Fig. S5. Treatment of spheroids with either the mock or HER2-CAR T cells. The ratio of the spheroids and either the mock or HER2-CAR T cells was 1:1 in the 3DHSP, and the treatment duration was 24 h. (A) Optical and fluorescent images of the treated spheroids of BT474, SKOV3, and U87 cells stained with calcein-AM (green) and ethidium homodimer-1 (red). (B) Area and green and red fluorescence intensity (C) of the treated spheroids. Scale bar: 200 μm. Student’s t-test: *p < 0.05, ***p < 0.001; NS, not significant. Fig. S6. Cytotoxicity of mock T and HER2-CAR T cells against luciferase-expressing cells co-cultured in 2D. The effector (BT474, SKOV3, and U87 cells) to target ratio was 4:1 (unless otherwise stated), and the cytotoxicity was assessed by the luminescence of surviving [file 12951_2021_1213_MOESM1_ESM.docx]

**Additional file 1**

3D hanging spheroid plate for high-throughput CAR T cell cytotoxicity assay

Zhenzhong Chen^1+^, Seokgyu Han^1+^, Arleen Sanny^2^, Dorothy Leung-Kwan Chan^2^, Danny van Noort^3,4^, Wanyoung Lim^5^, Andy Hee-Meng Tan^2,*^, and Sungsu Park^1,5,6,*^

^1^School of Mechanical Engineering, Sungkyunkwan University (SKKU), Suwon 16419, Korea

^2^Bioprocessing Technology Institute (BTI), Agency for Science, Technology and Research (A*STAR), Singapore 138668, Singapore

^3^Centro de Investigación en Bioingeniería, Universidad de Ingenieria y Tecnologia - UTEC, Lima 04, Peru

^4^Biotechnology, Linköping University, SE-581 83 Linköping, Sweden

^5^Department of Biomedical Engineering, Sungkyunkwan University (SKKU), Suwon 16419, Korea

^6^Institute of Quantum Biophysics (IQB), Sungkyunkwan University (SKKU), Suwon 16419, Korea

^+^ These authors contributed equally to this work.

^*^**Corresponding authors:** A. Tan, Bioprocessing Technology Institute (BTI), Agency for Science, Technology and Research (A*STAR), Singapore. [Tel: +65-6-407-0937](mailto:Tel:%20+65-6-407-0937). E-mail: andy_tan@bti.a-star.edu.sg/S. Park, School of Mechanical Engineering, Sungkyunkwan University (SKKU), Suwon 16419, Korea. Tel: +82-31-290-7431. E-mail: [nanopark@skku.edu](mailto:nanopark@skku.edu).


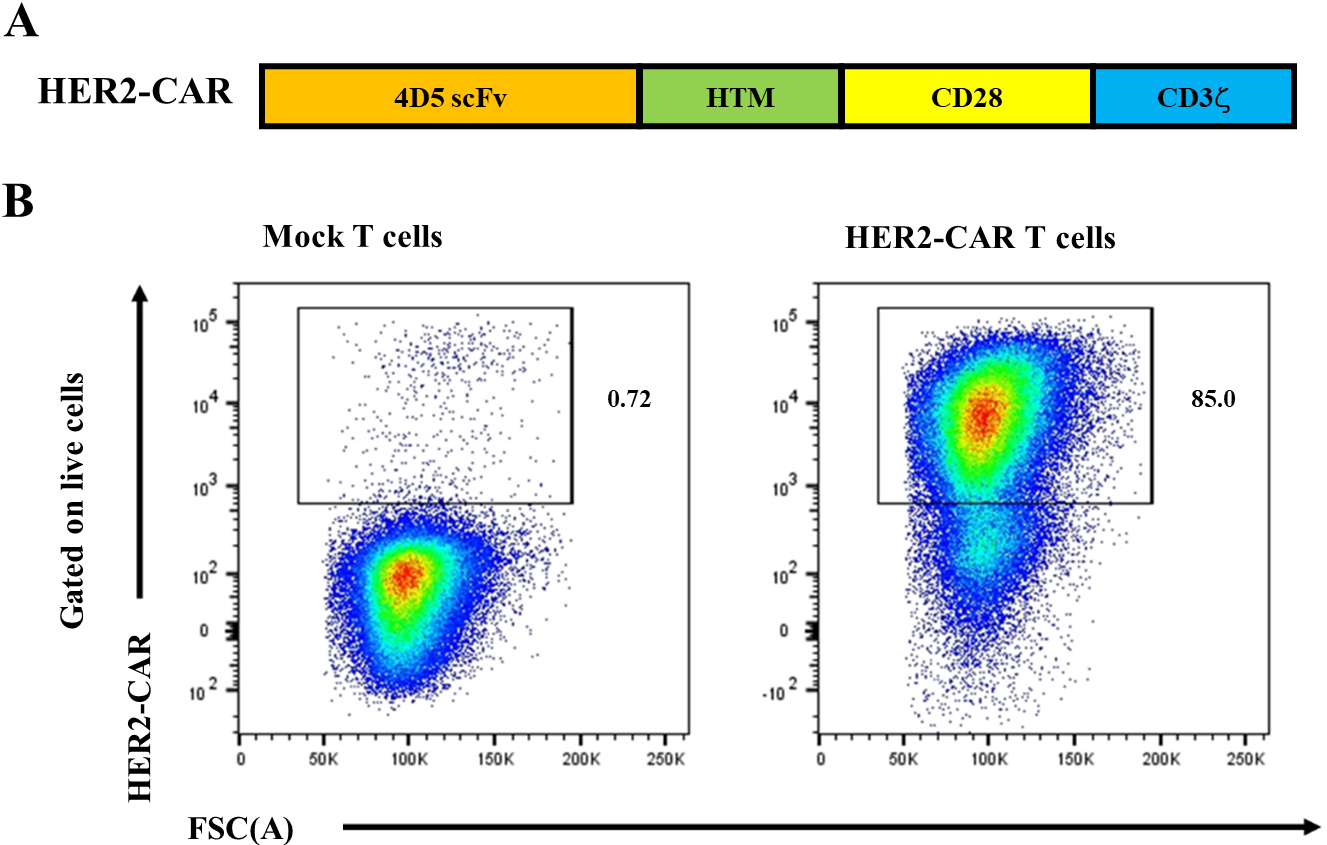


**Fig. S1.**Generation of human epidermal growth factor receptor 2 (HER2)-chimeric antigen receptor (CAR) T cells. (A) Schematic diagram showing HER2-CAR structure. (B) Frequency of HER2-CAR T cells as assessed using flow cytometry. Data in (B) are representative of three independent experiments: HTM, hinge and transmembrane region.


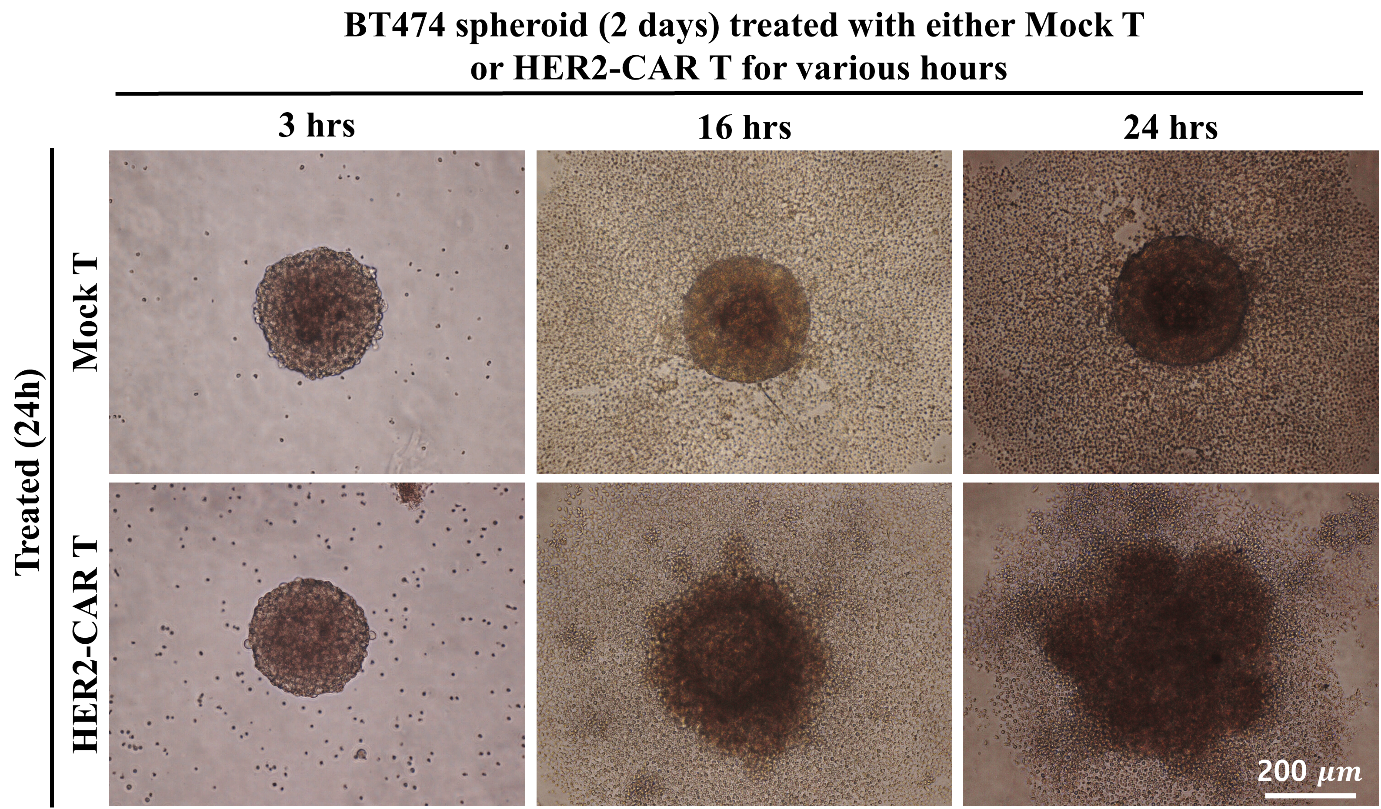


**Fig. S2.** Treatment of BT474 spheroids with either mock T or HER2-CAR T cells. The ratio of BT474 spheroids to either mock T or HER2-CAR T cells was 1:4 in agarose-coated wells, and the treatment duration was 3, 16, and 24 hrs.

.


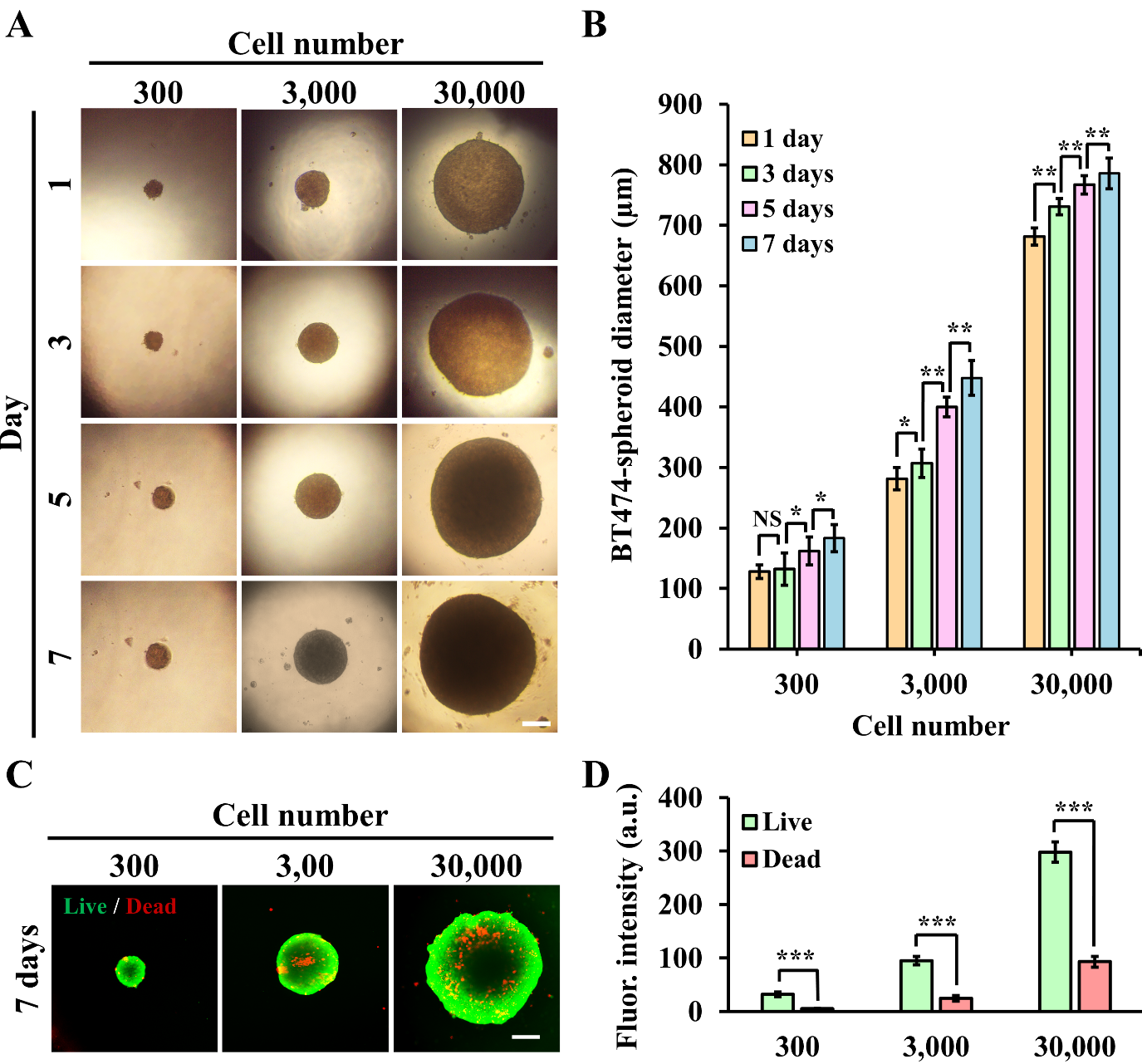


**Fig. S3.** Formation of BT474 spheroids on the 3DHSP. (A) Micrographs of BT474 spheroids with different cell numbers (300, 3,000, and 30,000) on the 3DHSP for 1, 3, 5, and 7 d. (B) Spheroid diameter changing with time. (C) Representative images of BT474 spheroids cultured on the 3DHSP for 7 d showing the live (green) and dead (red) cells. (D) Green and red fluorescence intensity of spheroids cultured on the 3DHSP for 7 d. Scale bar: 200 µm. Student’s t-test: * p< 0.05, ** p< 0.01, *** p< 0.001; NS, not significant.


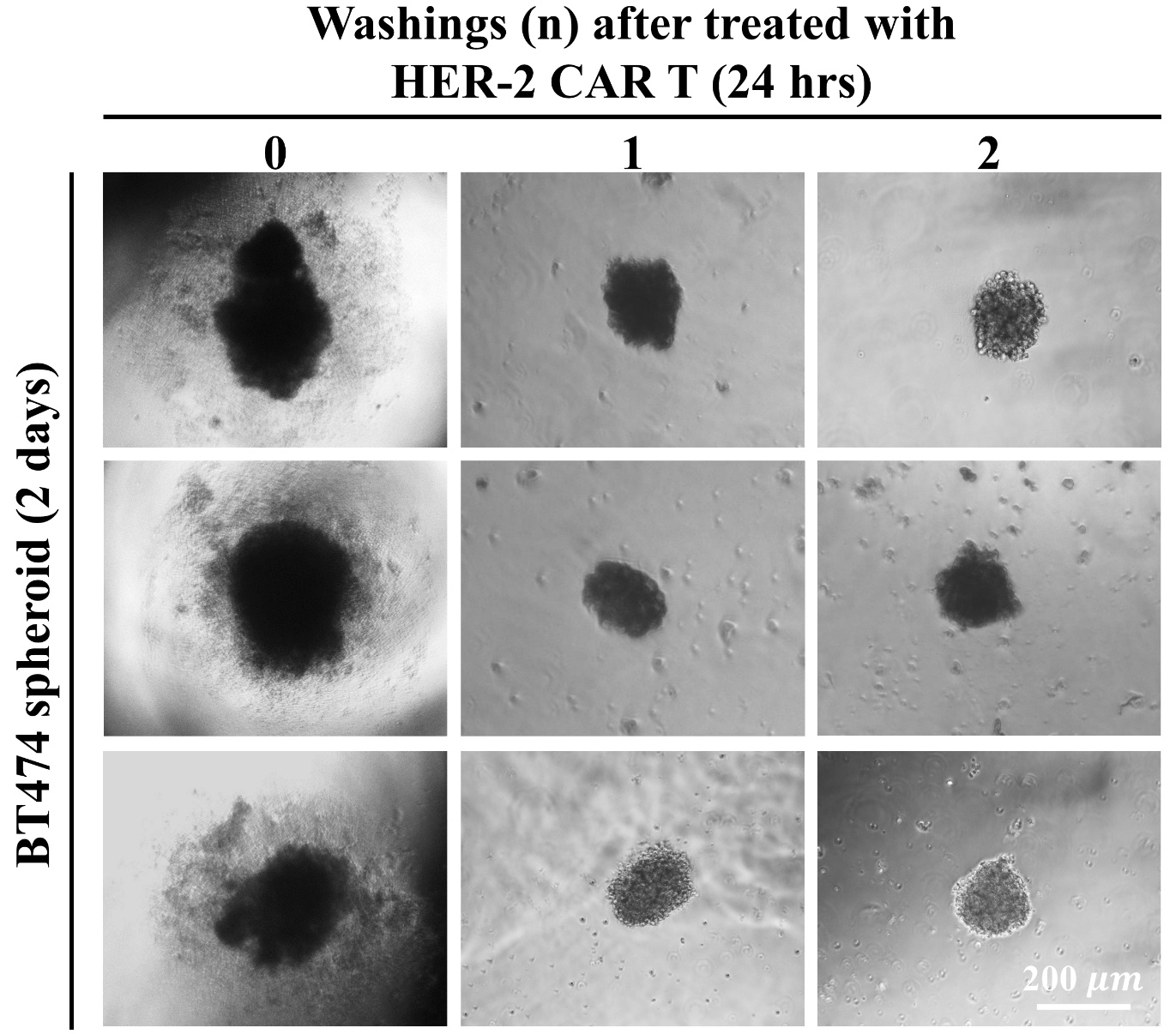


**Fig. S4.** Image of the treated BT474 spheroids with and without either one or two repeated washings. Scale bar: 200 µm.


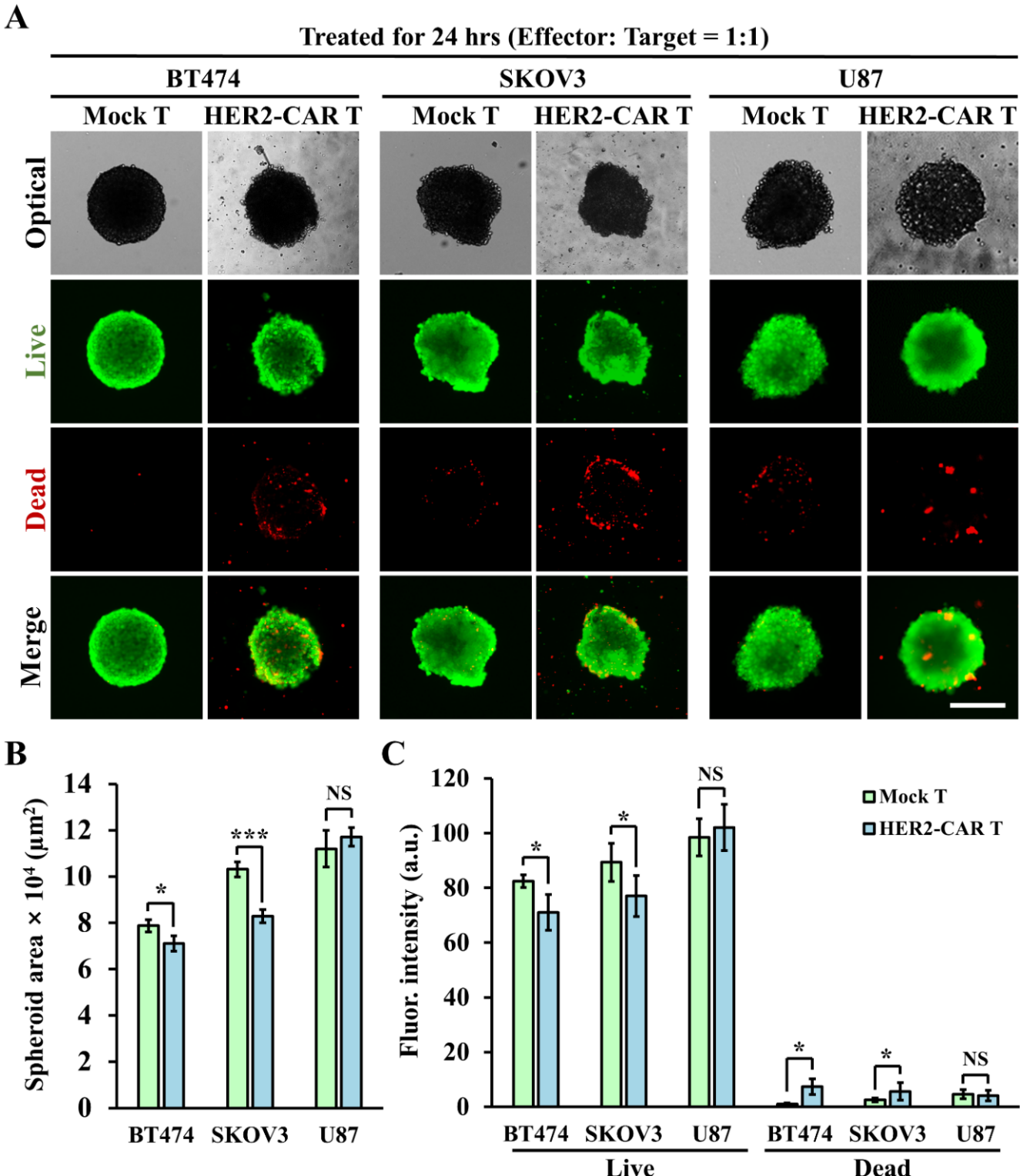


**Fig. S5.** Treatment of spheroids with either the mock or HER2-CAR T cells. The ratio of the spheroids and either the mock or HER2-CAR T cells was 1:1 in the 3DHSP, and the treatment duration was 24 hrs. (A) Optical and fluorescent images of the treated spheroids of BT474, SKOV3, and U87 cells stained with calcein-AM (green) and ethidium homodimer-1 (red). (B) Area and green and red fluorescence intensity (C) of the treated spheroids. Scale bar: 200 µm. Student’s t-test: * p< 0.05, *** p< 0.001; NS, not significant.

**
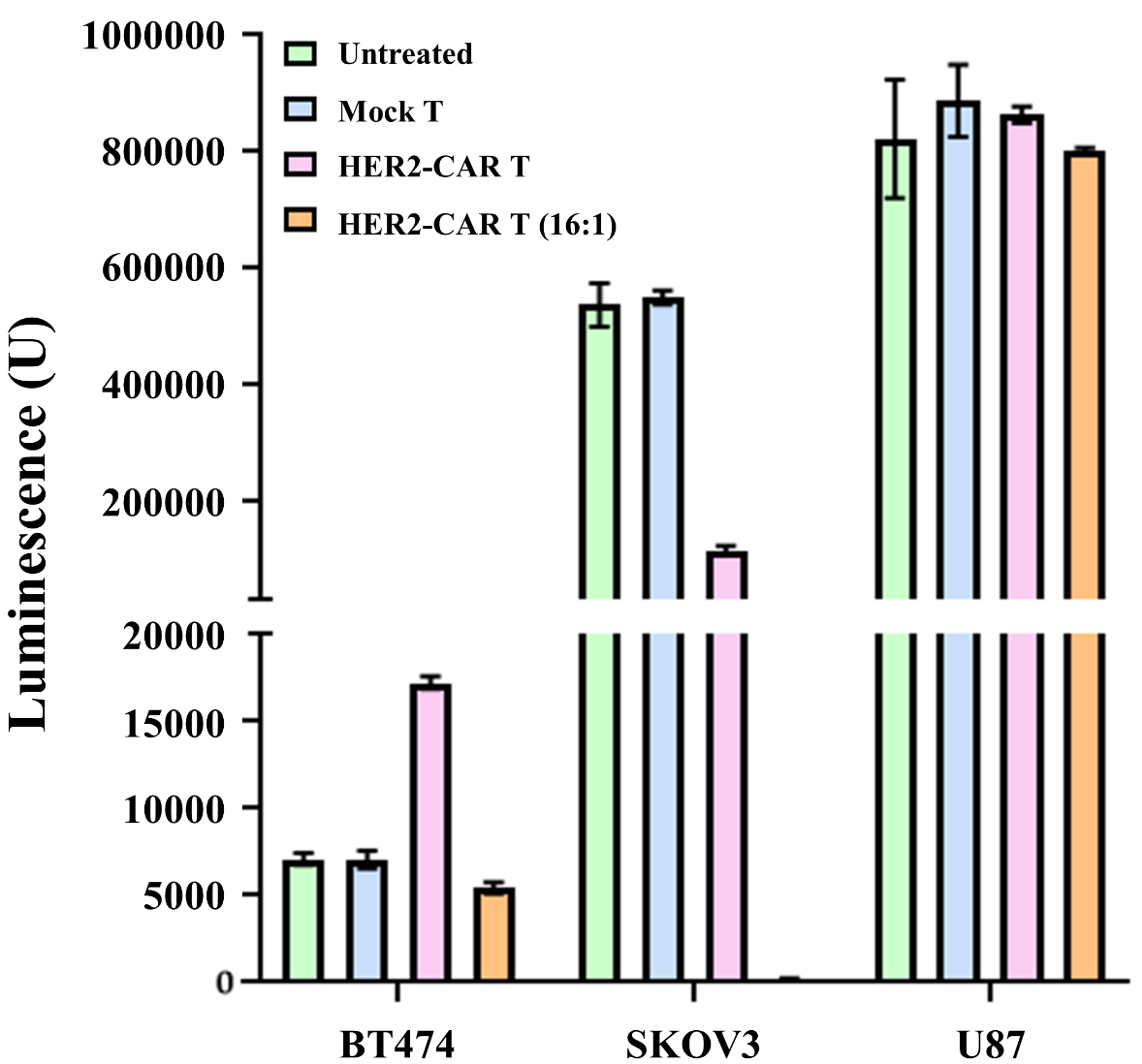
**

**Fig. S6.** Cytotoxicity of mock T and HER2-CAR T cells against luciferase-expressing cells co-cultured in 2D. The effector (BT474, SKOV3, and U87 cells) to target ratio was 4:1 (unless otherwise stated), and the cytotoxicity was assessed by the luminescence of surviving tumor cells 24 hrs later. Data are the mean ± standard deviation (SD) of three technical replicates for each condition and representative of one experiment.
